# Roemerine Improves the Survival Rate of Septicemic BALB/c Mice by Increasing the Cell Membrane Permeability of *Staphylococcus aureus*

**DOI:** 10.1371/journal.pone.0143863

**Published:** 2015-11-25

**Authors:** Sunjun Yin, Gaoxiong Rao, Jin Wang, Liyang Luo, Gonghao He, Chengying Wang, Chaoyu Ma, Xiaoxing Luo, Zheng Hou, Guili Xu

**Affiliations:** 1 Department of Pharmacy, Kunming General Hospital of Chengdu Military Region, Kunming, Yunnan, 650500, China; 2 Yunnan University of Traditional Chinese Medicine, Kunming, Yunnan, 650500, China; 3 Department of Pharmacology, School of Pharmacy, Fourth Military Medical University, Xi’an, Shaanxi, 710032, China; 4 Kunming Medical University, Kunming, Yunnan, 650500, China; Massachusetts General Hospital, UNITED STATES

## Abstract

*Staphylococcus aureus* is one of the most frequently occurring hospital- and community-associated pathogenic bacteria featuring high morbidity and mortality. The occurrence of methicillin-resistant *S*. *aureus* (MRSA) has increased persistently over the years. Therefore, developing novel anti-MRSA drugs to circumvent drug resistance of *S*. *aureus* is highly important. Roemerine, an aporphine alkaloid, has previously been reported to exhibit antibacterial activity. The present study aimed to investigate whether roemerine can maintain these activities against *S*.*aureus* in vivo and further explore the underlying mechanism. We found that roemerine is effective in vitro against four *S*. *aureus* strains as well as in vivo against MRSA insepticemic BALB/c mice. Furthermore, roemerine was found to increase cell membrane permeability in a concentration-dependent manner. These findings suggest that roemerine may be developed as a promising compound for treating *S*. *aureus*, especially methicillin-resistant strains of these bacteria.

## Introduction

*Staphylococcus aureus* (*S*. *aureus*) is one of the most prevalent pathogenic bacteria in hospitals and communities[1]. It is a major cause of infections worldwide and promotes many diseases such as skin infections, respiratory conditions (e.g., pneumonia), food poisoning, and bacteremia[2, 3]. Compared with bacteremia caused by other Gram-positive pathogens, *S*. *aureus* bacteremia is associated with high morbidity and mortality[4].

*S*. *aureus* infections are generally treated with antibacterial drugs. However, excessive exposure to antibacterial drugs has resulted in the development of drug-resistant strains of bacteria. In clinical settings, for example, vancomycin is the last line of treatment for multi-resistant infections caused by methicillin-resistant *S*. *aureus* (MRSA). However, vancomycin-resistant *S*. *aureus* (VRSA) has been observed with increasing frequency around the world[5]. Although linezolid was approved for clinical use in 2000 as the first commercially available oxazolidinone antibiotic, linezolid-resistant *S*. *aureus* was found in a patient merely one year later[6]. Thus, developing new anti-MRSA drugs with different chemical structures in order to address drug resistance is an urgent necessity.

Roemerine is an aporphine alkaloid that can be isolated from many plants such as Annona senegalensis[7], Turkish Papaver[8] and Rollinialeptopetala[9]. It was previously found that aroemerine derivative had antibacterial effects against three Gram-positive bacteria (namely, *Bacillus cereus*, *Micrococcus sp*., and *S*. *aureus*) with minimal inhibitory concentrations (MICs) in the range of 50–300 μg/mL[10]. In the past few decades, only a few studies have been performed on the antimicrobial activity of roemerine[7, 10]. The present study was thus undertaken to investigate the activity of roemerine against *S*. *aureus* in vivo as well as its underlying mechanisms.

## Materials and Methods

### Bacterial strains

*S*. *aureus* ATCC29213 was purchased from the Chinese National Center for Surveillance of Antimicrobial Resistance. Strain Mu50 (ATCC700699) was purchased from MicroBiologics (Saint Cloud, MN, USA). Strain LAC (USA300) was a generous gift from Michael Otto (NIH). MRSA XJ75302 was obtained from the clinical laboratory of Xijing Hospital (Xi’an, China). Fifteen MRSA strains were obtained and characterized from the infectious sputum of critically ill patients in Kunming General Hospital.

### Antibacterial agents and chemicals

All of the antimicrobial agents were purchased from their respective manufacturers in China, i.e., oxacillin was obtained from CSPC Zhongnuo Pharmaceutical Co., Ltd; linezolid was obtained from Pfizer Inc.; vancomycin was obtained from Eli Lilly Japan K. K., Seishin Laboratories; amikacin was obtained from Jiangsu Wuzhong Pharmaceutical Group Co., Ltd; furantoin was obtained from Shanxi Yunpeng Pharmaceutical Co., Ltd; netilmicin was obtained from Guangdong Luofushan Pharmaceutical Co., Ltd; fosfomycin was obtained from Northeast Pharmaceutical Group Daiichi Pharmaceutical Co., Ltd Shenyang; cefoperazone sulbactam was obtained from Pfizer Inc.; and levofloxacin was obtained from Jiangsu Yangtze River Pharmaceutical Co., Ltd.

Roemerine (purity 97%) was kindly provided by Professor Gaoxiong Rao of the School of Pharmacy, Yunnan College of Traditional Chinese Medicine[11]. Dis-C_3_-(5) was purchased from Molecular Probes Co. LIVEDEAD Backlight Bacterial Viability Kit was purchased from Invitrogen Corp. All other chemicals used were purchased from Sigma-Aldrich Co. Ltd.

### Susceptibility testing

MICwas determined by the standardized broth microdilution technique with starting inocula of 5×10^5^ colony forming units (CFU)/mL as recommended by the Clinical Laboratory Standards Institute (CLSI). Agents were added at a concentration range of 0.25–1024μg/mL; equal volumes of MRSA cultures were set as controls. The lowest concentration inhibiting visible bacterial growth was recorded as the MIC. Minimum bactericidal concentration (MBC) were determined by plating 100 μL samples obtained from wells with no visible turbidity onto Mueller-Hinton agar plates. The lowest concentration showing growth of less than five colonies on agar subculture was taken as the MBC.

Roemerine was added to each tested strain culture to a final concentration of 0, 8, 16, 32 or 64μg/mL. The strains were cultivated in an automated Bioscreen C system (Lab systems Helsinki, Finland) using MH broth culture medium. The working volume in the wells of the Bioscreen plate was 300 μLwith150 μL of MH broth and 150 μL of drug solution. The temperature was controlled to 37°C, and the optical density of the cell suspensions was measured automatically at 600 nm every 1 h for 20 h.

The time-kill curves for four *S*. *aureus* strains were determined by using the drop plate method[12]. The number of colony-forming units (CFUs) was calculated from the number of colonies growing on the plates. Compounds of different concentrations were added to the cell cultures. Aliquots of each culture were collected at 0, 3, 6, 12, or 24h, diluted, and inoculated on solid agar. Plates were then incubated for 48 h at 37°C.

### Antibacterial activity in vivo

BALB/c mice (5–6 weeks old, weighing 18–22 g) were obtained from the Animal Center of Fourth Military Medical University (Xi’an, China). The mice were housed under controlled ambient conditions (12 h light/dark cycle) and were allowed free access to a standard rodent diet and distilled water. A period of 4 days was allowed for the animals to acclimatize before any experimental manipulations were undertaken.

BALB/c mice were divided into four groups with 17 mice per group. A50 mg/mL solution of pentobarbital in sterile saline was administered via intraperitoneal (i.p.) injection at a dose of 50 mg/kg. Then BALB/c mice were then infected by intravenous (i.v.) injection of 0.1 mL (1.0×10^10^CFU/mL) of MRSAXJ75302 under anesthesia for 15 minutes. After bacterial challenge for 1 h, mice were randomized to receive i.p. injection of roemerine, oxacillin, or vancomycin at 20 mg/kg once daily for 2 d. The model group received i.p. injection with an equivalent volume of saline. To assess bacterial clearance, 5 mice in each group were removed from their cages 8 h after the last drug administration and gently restrained on the benchtop. Cervical dislocation was performed manually and resulted in euthanasia within 10 seconds. A total of 100 μl blood from heart and tissues (blood, kidney, heart, liver) were rapidly collected aseptically from each animal, weighed, and homogenized in sterile saline solution. Bacterial counts were determined in these tissues and expressed as CFUs/gram of tissue.

The survival of 12 mice in each group was monitored for 7 d after infection, and the cumulative percentage survival was determined. Mice were monitored every 8 hours for the first 48 hours and then twice daily for the remaining five days. The criteria used for determining the pre-moribund state included development of a hunched posture, decreased activity, labored breathing, loss of appetite, and piloerection[13]. If mice were found in the pre-moribund state, they were euthanized with carbon dioxide, and death was verified be monitoring for cardiac cessation and respiratory arrest.

### Transmission electron microscopy

*S*. *aureus* ATCC29213 (1×10^7^ CFU/mL) were cultured with 0, 32, 48 or 64 μg/mL roemerine at 200 rpm and at 37°C for 8 h, harvested, and then washed[14]. The specimens were observed by transmission electron microscopy (TEM)(JEM-1230, JOEL), and relevant images were recorded.

### Cell membrane permeability assay

*S*. *aureus* ATCC29213 (5×10^6^ CFU/mL) was cultured with 32, 64, or128μg/mL roemerine at 200 rpm and 37°C for 6h; the control group was treated without drugs under the same conditions. Bacteria were harvested, washed, stained with Syto 9 and propidium iodide (BacLight Bacterial Viability kit; Molecular Probes)[15], and then examined by H600L fluorescence microscopy (Nikon, Japan).

### Cytoplasmic membrane depolarization assay

Cytoplasmic membrane depolarization of roemerine was determined with the membrane potential-sensitive dye dis-C_3_-(5)[16, 17]. *S*. *aureus* ATCC29213 in the mid-logarithmic phase was suspended in 5 mM HEPES (pH 7.2) to yield an optical density of 0.05 at 630 nm. Then, dis-C_3_-(5) was added to the cells at a final concentration of 100 nM. Fluorescence was quenched at 25°C for 15 min. About 1mL of this suspension was placed in a 1cm cuvette. Roemerine was subsequently added to this cuvette to achieve a final concentration of 0, 32, 64 or 128 μg/mL. Changes in fluorescence were monitored using an F-2500 fluorescence spectrophotometer (Hitachi, Japan) at excitation and emission wavelengths of 622 and 670 nm, respectively.

In a modified form of the assay described above [17], *S*. *aureus* ATCC29213 was treated with no antibiotic (control), or64 or 128 μg/mL roemerine at 37°C. 1mL *S*. *aureus* was collected at 0, 15, 30, 60, 90 min and incubated with dis-C_3_-(5) (100 nM final concentration) for an additional 15 min at 25°C. Then, samples were transferred to cuvettes, cells were excited at 622 nm, and fluorescence emission was collected at 670 nm for 10 s. Time-kill curves for *S*. *aureus* ATCC29213 were determined by the drop plate method as mentioned above.

### Ethics statement

All clinical strains were collected from patients. The study was approved by the Ethics Committee of the Fourth Military Medical University and the Kunming General Hospital of Chengdu Military Region. Written informed consent was not obtained, but patient record/information was anonymized and de-identified prior to analysis. Both the Ethics Committee of the Fourth Military Medical University and the Kunming General Hospital of Chengdu Military Region specifically approved the lack of informed consent in this study.

All animal experiments were carried out in strict accordance with the recommendations in the Guide for the Care and Use of Laboratory Animals of the Fourth Military Medical University (Xi’an, China). The study was approved by the Committee on the Ethics of Animal Experiments of the Fourth Military Medical University.

### Statistical analyses

**Fig 1:**
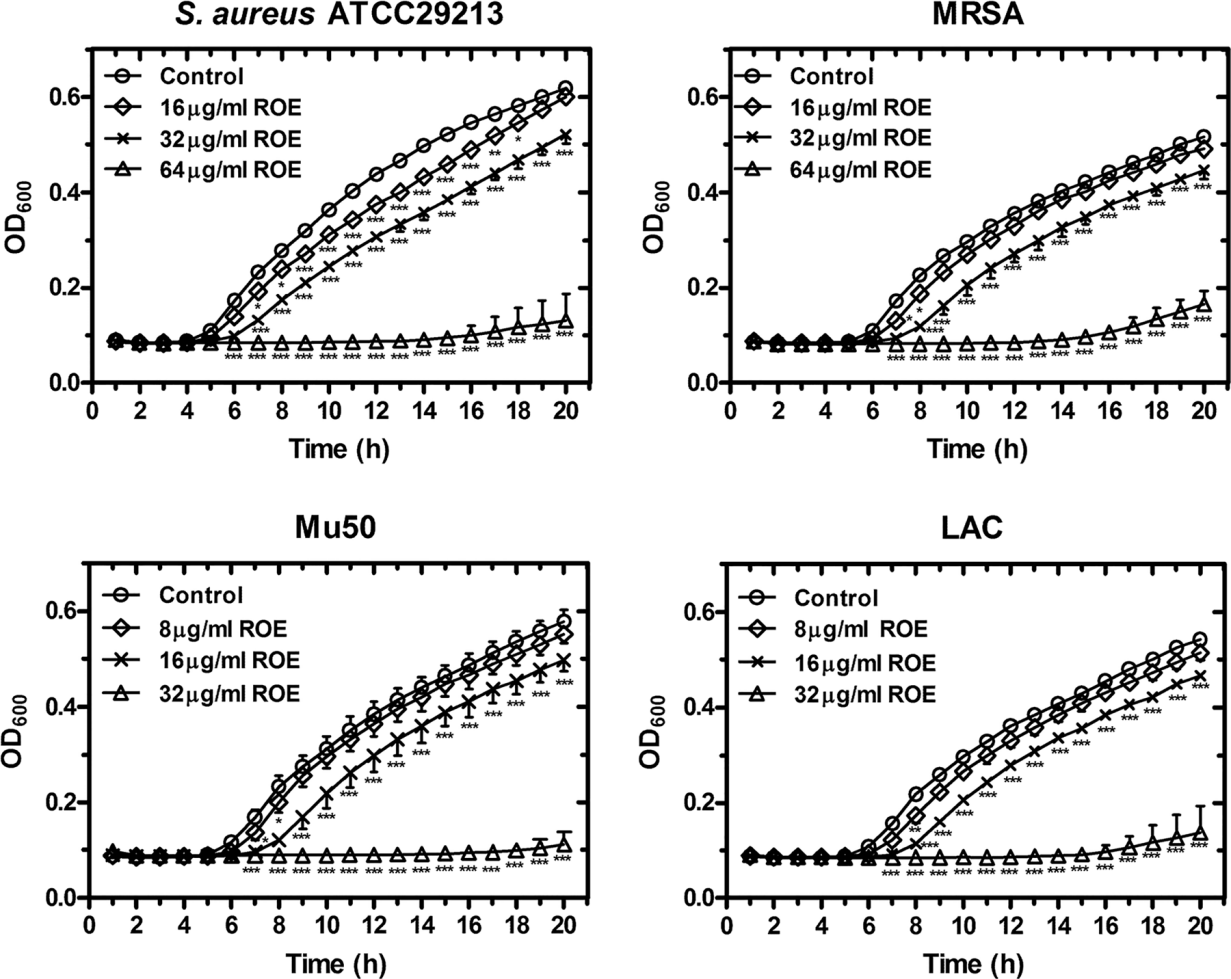
Concentration-dependent inhibition of roemerine on the growth of four *S*. *aureus*. Roemerine was added to cell cultures containing different tested strains to a final concentration 8, 16, 32, or 64 μg/mL, with addition of an equal volume of sterile water as a control. The growth curves for four tested strains were measured using a BioscreenC™ instrument in the absence and presence of different concentrations of roemerine. The sample frequency was one hour and data at time points are the means for three replicates. ROE, roemerine. *P<0.05, **P<0.01, ***P<0.001 versus Control.

**Fig 2:**
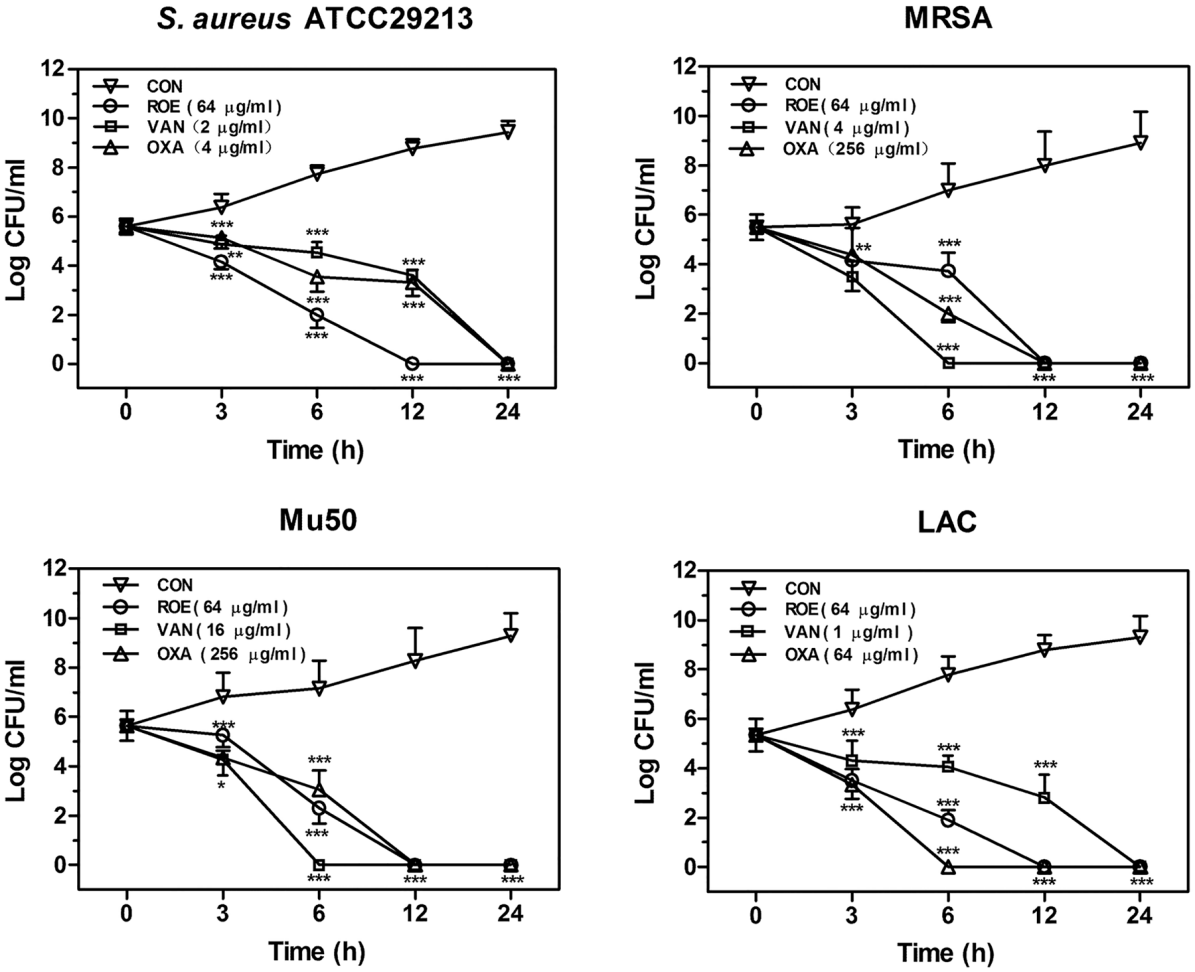
Effects of roemerine on the growth of bacteria colonies. Roemerine, vancomycin and oxacillin were added to cell cultures containing *S*. *aureus* ATCC29213, MRSA XJ75302, Mu50, or LAC, respectively, with addition of an equal volume of sterile water as a control. Aliquots of each culture were collected at 0, 3, 6, 12, and 24 h, and were then diluted and inoculated on solid agar. The number of colony-forming units (CFUs) was calculated from the number of colonies growing on plates, and the data are the means for three replicates. CON, control; ROE, roemerine; VAN, vancomycin; OXA, oxacillin. *P<0.05, **P<0.01, ***P<0.001 versus Control.

**Fig 3:**
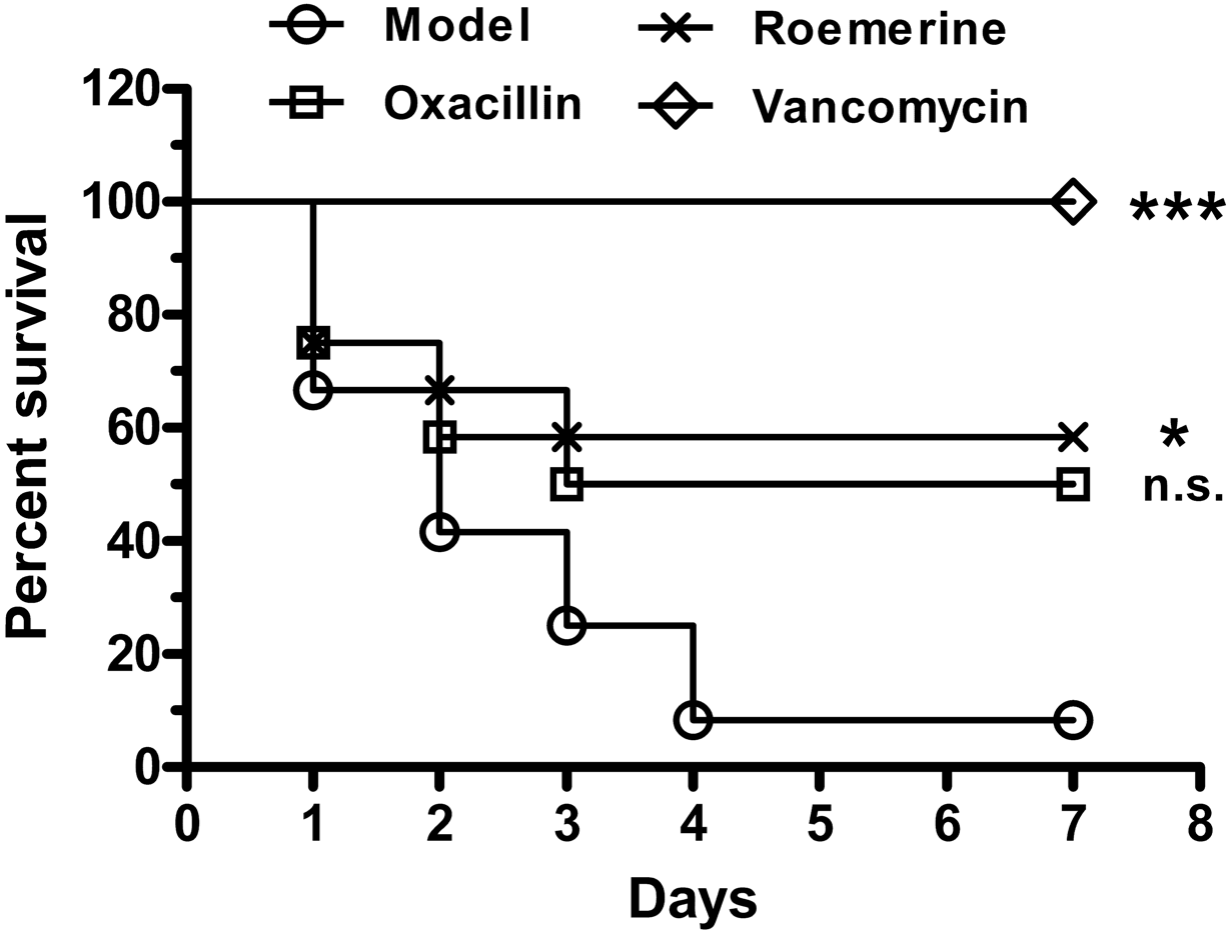
Activity of roemerine against MRSA XJ75302 in a mouse sepsis model. Survival of BALB/c mice inoculated by i.p. injection with MRSA XJ75302 and treated with 20 mg/kg roemerine, oxacillin, vancomycin or sterile water by i.p. administration. Values with statistical significance by the log-rank test are indicated by an asterisks: *P<0.05, ***P<0.001 versus Model group.

**Fig 4:**
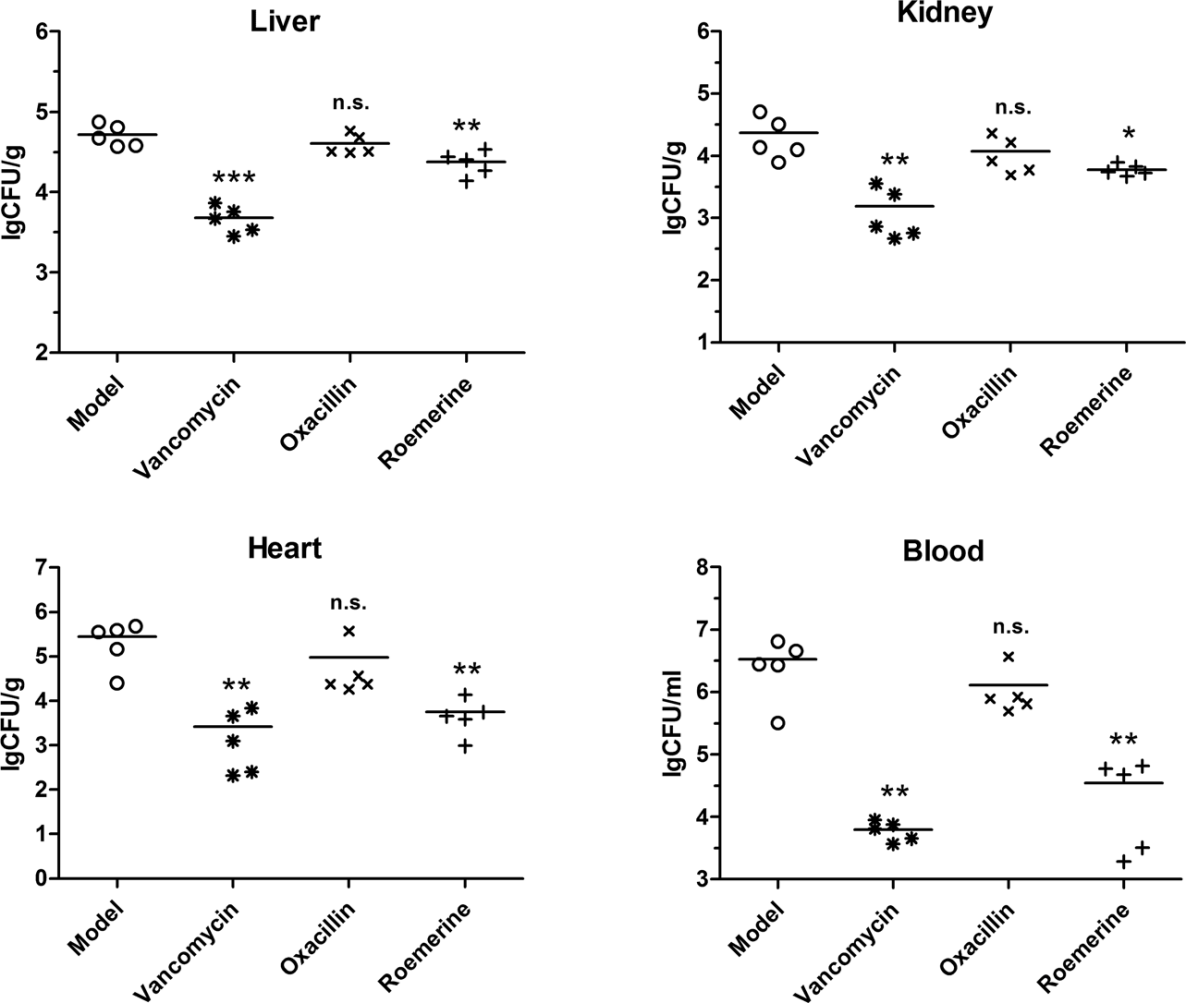
Colonization of *S*. *aureus* inoculum in the liver, kidney, heart and blood of roemerine- or vancomycin-treated BALB/c mice (CFUs/gram of tissue). *P<0.05, **P<0.01, ***P<0.001 versus Model.

**Fig 5:**
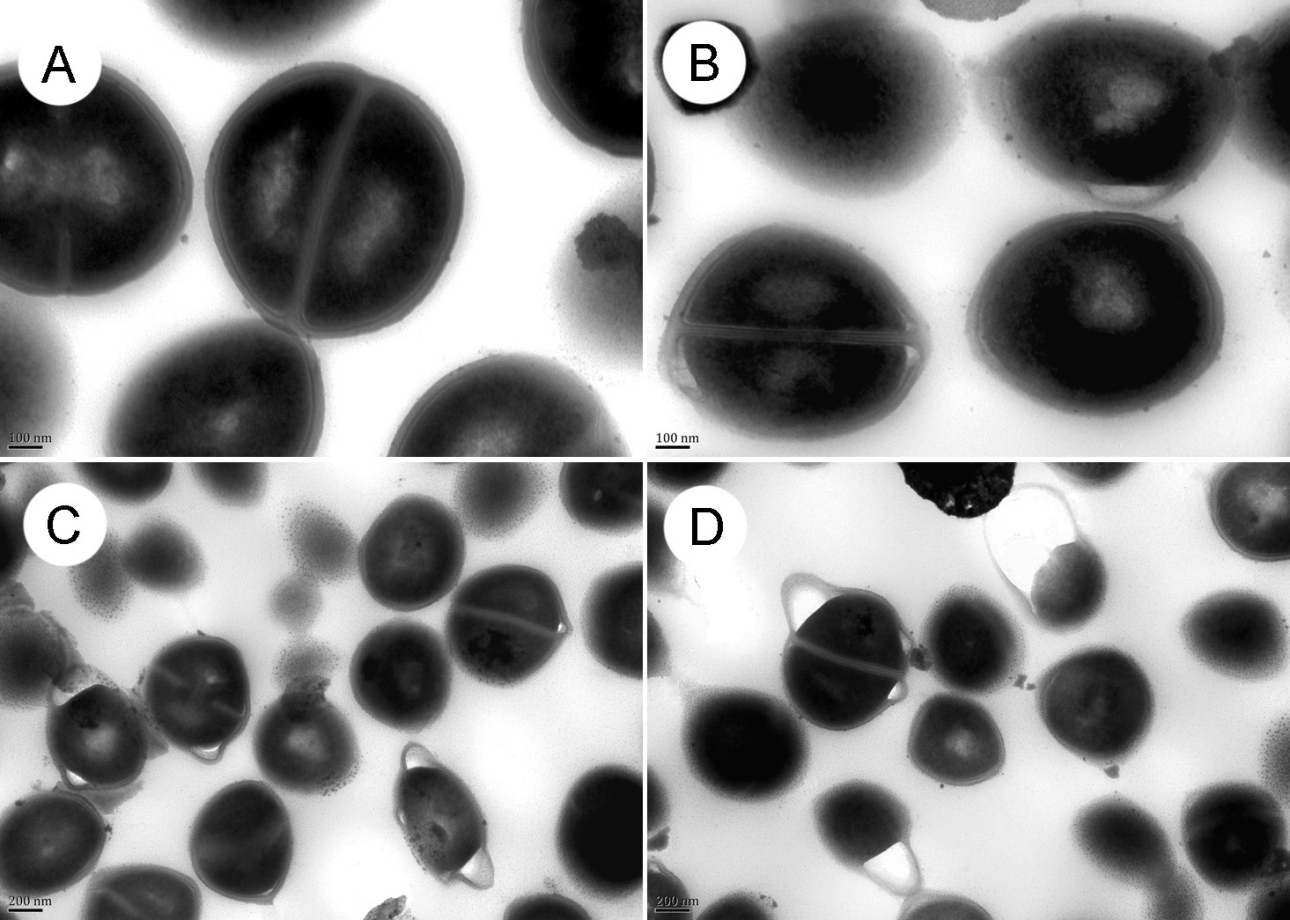
The morphology of *S*. *aureus* ATCC29213 was investigated by transmission electron microscopy after treatment with 0, 32, 48 or 64 μg/mL roemerine for 8 h. A, Control; B, *S*. *aureus* ATCC29213 treated with 32 μg/mL roemerine; C, *S*. *aureus* ATCC29213 treated with 48 μg/mL roemerine; D, *S*. *aureus* ATCC29213 treated with 64 μg/mL roemerine. A and B, ×100K; C and D, ×50K.

**Fig 6:**
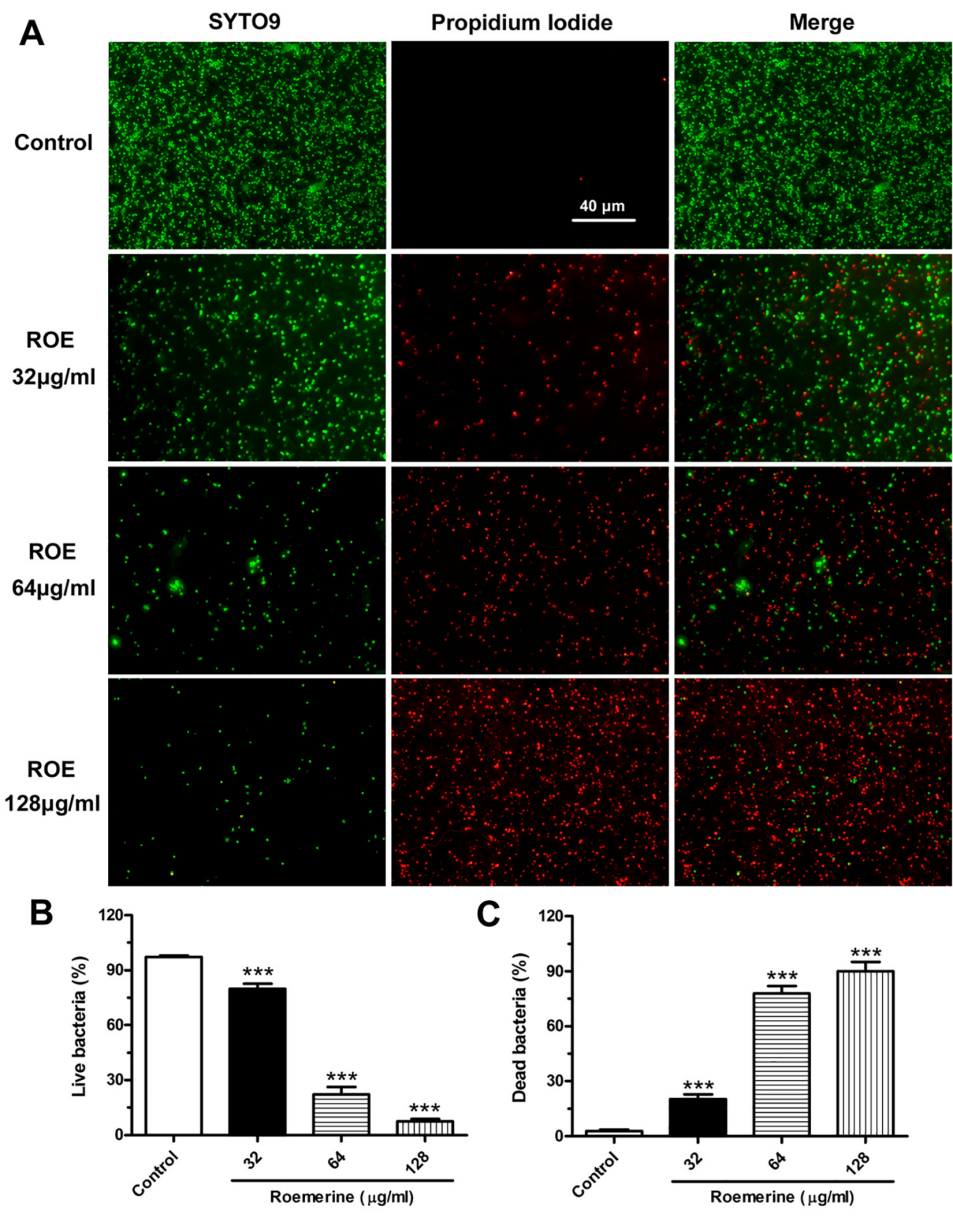
Roemerine-mediated bacterial membrane permeabilization (original magnification, ×1000). (A) Cultured *S*. *aureus* ATCC29213 was treated with 0, 32, 64 or 128 μg/mL of roemerine for 6 h, then were stained with the dyes SYTO 9 (which stains live bacteria) and propidium iodide (which stains dead bacteria). The stained bacteria were analyzed by fluorescence microscopy to assess viability. (B) The numbers indicate the percentages of live bacteria within *S*. *aureus* ATCC29213 after treatment with different concentrations of Roemerine. (C) The numbers indicate the percentages of dead bacteria within *S*. *aureus* ATCC29213 after treatment with different concentrations of Roemerine.

**Fig 7:**
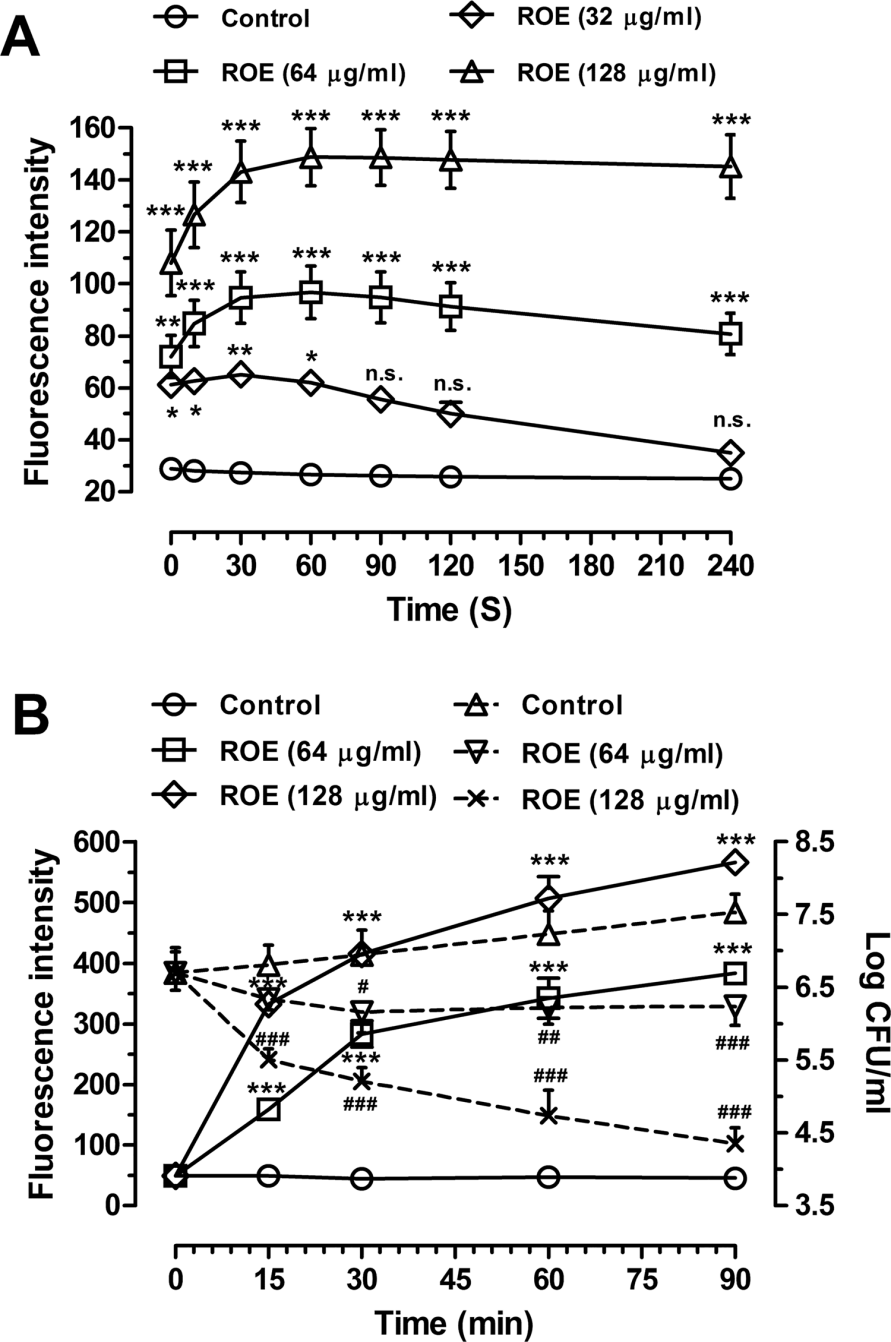
Cytoplasmic membrane depolarization of *S*. *aureus* ATCC29213. (A) Cytoplasmic membrane depolarization of *S*. *aureus* treated with roemerine after dis-C_3_-(5) dye incubation. Control (circles), 32 μg/mL ROE (diamond), 64 μg/mL ROE (square), 128 μg/mL ROE (triangle). ***P<0.001 versus Control. (B) Cytoplasmic membrane depolarization of *S*. *aureus* treated with roemerine before dis-C_3_-(5) dye incubation. Roemerine treatment (Left Y axis) and corresponding time-kill curves (Right Y axis). Left Y axis: Control (circles with solid line), 64 μg/mL ROE (square with solid line), 128 μg/mL ROE (diamond with solid line). Right Y axis: Control (triangles with dash line), 64 μg/mL ROE (inverted triangle with dash line), 128 μg/mL ROE (X-type with solid line).ROE indicate roemerine. ^#^ *P*<0.05, ^##^ *P*<0.01, ^###^ *P*<0.001 versus Control.

Data are expressed as means ± SD in Figs 1–6 and 7B. Data are shown as mean ± SEM in Fig 7A. Comparisons of three or more groups were performed using one-way ANOVA (Figs 4 and 6). Two-way ANOVA was used in Figs 1, 2 and 7. Survival curves were calculated by the Kaplan-Meier method (Fig 3). Statistical analyses were done using the Prism 6 software (Graphpad, CA). *P*≤0.05 was considered to be statistically significant.

## Results

### Growth assay

Roemerine exhibited bactericidal effects against all tested *S*. *aureus* strains (both methicillin-susceptible and methicillin-resistant strains) with MICs ranging from 32 μg/mL to 64 μg/mL (Table 1). The MBCs of roemerine against *S*. *aureus* were either one- or two-fold higher than the MICs (Table 1). As shown in Fig 1, roemerine completely inhibited the growth of four *S*. *aureus* strains at concentrations of 32 or 64 μg/mL. Kill curves indicated that 64 μg/mL roemerine reduced the bacterial population from nearly 5×10^5^ CFUs/mL to <20 CFUs/mL within 24 h. The time-kill curves of vancomycin and oxacillin were similar to that of roemerine. In the control groups, however, increases in the concentrations of four *S*. *aureus* strains of about 10^5^CFUs/mL were observed within 24 h (Fig 2).

**Table 1:** MICs of roemerine and antibiotics in Mueller-Hinton broth culture.

Strains	MIC (μg/mL)										MBC (μg/mL)
	ROE	OXA	LZD	VAN	AMI	FUR	NET	FOS	CFP	LVF	
*S*. *aureus*ATCC29213	64	0.5	2	1	2	16	0.25	2	2	0.25	64
LAC USA300	32	32	—	—	—	—	—	—	—	—	64
Mu50	32	256	—	—	—	—	—	—	—	—	64
MRSA XJ75302	64	128	—	—	—	—	—	—	—	—	64
MRSA 8	32	64	2	0.5	16	16	<0.25	16	1024	64	32
MRSA 15	64	128	1	1	32	16	0.25	32	1024	64	64
MRSA 68	32	64	1	1	32	32	4	32	1024	64	64
MRSA 82	32	32	1	1	32	32	4	32	1024	64	64
MRSA 92	64	32	1	1	16	16	4	32	1024	64	64
MRSA 98	64	32	1	1	32	16	4	64	1024	64	64
MRSA 144	32	32	1	0.5	32	32	4	64	512	64	64
MRSA 153	64	64	1	0.5	16	32	4	32	512	64	64
MRSA 202	64	128	1	0.5	32	32	4	32	1024	64	128
MRSA 214	32	64	2	1	32	32	4	64	512	64	64
MRSA 234	32	32	1	1	32	16	4	32	1024	32	64
MRSA 237	32	64	0.5	1	64	32	4	32	512	32	64
MRSA 246	64	64	2	1	64	16	4	32	1024	32	64
MRSA 310	32	64	2	1	32	32	2	32	1024	32	64
MRSA 321	64	128	2	2	32	16	4	64	1024	64	64

ROE, OXA, LZD, VAN, AMI, FUR, NET, FOS, CFP and LVF indicate roemerine, oxacillin, linezolid, vancomycin, amikacin, furantoin, netilmicin, fosfomycin, cefoperazone sulbactam, and levofloxacin, respectively.“–” showed that MIC test not done.

### In vivo antibacterial activity

Therapeutic effects of roemerine and vancomycin were observed in septicemic mice infected by MRSA XJ75302. The survival rates for roemerine and oxacillinat 20 mg/kg were 58.3% and50.0%, respectively. None of the infected mice died in the 20 mg/kg vancomycin treatment group (Fig 3). However, the survival rate of the model group was only 8.3% within 7 d of infection. Significant differences were observed between the 20 mg/kg roemerine and model groups (*P*<0.05) (Fig 3).

Since survival is related to bacterial titers in mice tissues, CFUs were measured among the different treatment groups. In the 20 mg/kg roemerine treatment group, the colony count (lg CFU/mL) in liver tissue decreased from 4.71 to 4.38, while that in kidney tissue decreased from 4.37 to 3.79, that in the heart decreased from 5.44 to 3.76, and that in the blood decreased from 6.52 to 4.55 (Fig 4). The group treated with 20 mg/kg vancomycin showed potent protective effects and CFU counts in tissue samples of this group decreased significantly (Fig 4).

### Ultrastructural analysis of bacterial cell walls

TEM images showed that *S*. *aureus* ATCC29213 cells developed well with intact cell walls in the control group without roemerine treatment (Fig 5A). By contrast, the cell walls of *S*. *aureus* ATCC29213 showed obvious bulges and separation from the cell membrane after roemerine treatment(Fig 5B, 5C and 5D).

### Bacterial membrane permeabilization

Bacterial membrane permeability was investigated by using membrane-permeant green fluorescent SYTO 9 and membrane-impermeant red propidium iodide. Under a microscope, cells of the control group showed nearly complete green fluorescence (Fig 6). As roemerine concentration increased, the number and intensity of red fluorescence spots gradually increased. Roemerine caused the number of green fluorescence spots to decrease in a concentration-dependent manner (Fig 6). These phenomena indicate that roemerine causes obvious increase in bacterial cell membrane permeability.

### Bacterial cytoplasmic membrane depolarization

Dis-C_3_-(5) was released after roemerine treatment in a concentration-dependent manner (Fig 7A), with high concentrations of roemerine releasing more dis-C_3_-(5) compared to low concentrations (Fig 7A). Stronger outcomes were observed with roemerine treatment before dis-C_3_-(5) dye incubation (Fig 7B), which indicated that roemerine can cause destabilization of the cytoplasmic membrane. The maximum fluorescence achieved was 383.1 and 566.5 arbitrary units by 64 and 128 μg/ml roemerine treatment, respectively, for 90 min (Fig 7B). Meanwhile, an inverse relationship was observed between the number of *S*. *aureus* CFUs and the amount of dis-C_3_-(5) released (Fig 7B).

## Discussion

Bacterial resistance has become increasingly severe in the world due to the widespread use of antibiotics. Currently, only a few new drugs target Gram-positive bacteria and most drugs are well-known antibacterial class analogues. Use of these drugs will inevitably induce cross-resistance in Gram-positive bacteria[18]. Therefore, novel methods and compounds for antibacterial treatment are being investigated, such as photodynamic therapy (PDT)[19, 20], and compounds with new structure and novel mechanisms of action[21, 22].

Susceptibility testing showed that roemerine displays similar antimicrobial activities toward methicillin-resistant *S*. *aureus* and on methicillin-susceptible *S*. *aureus*. Results of our in vitro bacterial killing assay are also consistent with this finding. Growth of *S*. *aureus* was completely inhibited after incubation for 12h with roemerine at 64 μg/mL, which is very similar to the effect of vancomycin and oxacillin.

Some studies have been done to investigate roemerine plasma pharmacokinetics and tissue distribution in rats by LC–MS/MS[11]. Results showed that roemerine had a *t*_1/2_ of 1.77±0.41 h in rats following i.v. administration at 6 mg/kg, with a C_max_ of 1835±214 μg/L[11]. Based on these data, in vivo experiments were carried out using a septicemic BALB/cmouse model. Results showed an obvious increase in the survival rate of septicemic mice after 20 mg/kg roemerine treatment. Moreover, roemerine also decreased bacterial loads in the liver, kidney, blood, and heart. All of these in vivo data indicate that roemerine can exert protective effects in septicemic BALB/c mice.

TEM assay revealed severe bulging and separation of cell wall from the cell membrane in *S*. *aureus* ATCC29213 treated with roemerine. Moreover, the gap between the cell wall and cell membrane grew bigger with increasing concentration of roemerine (Fig 5B, 5C and 5D). Cell membrane permeability assay showed more red fluorescence and less green fluorescence in *S*. *aureus* ATCC29213 cells after treatment with increasing concentrations of roemerine (Fig 6). These observations indicate gradual enhancement of membrane permeabilization after roemerine treatment.

Roemerine was also found to induce membrane depolarization in *S*. *aureus* ATCC29213. The cell membrane of *S*. *aureus* ATCC29213 depolarized, release of dis-C_3_-(5) increased, and a consequent increase in fluorescence was observed in all roemerine treatment groups (Fig 7). With increase in roemerine concentration, the number of live bacteria decreased and membrane depolarization increased. Taken together, these data indicate that roemerine caused *S*. *aureus* cell death by increasing cell membrane permeability.

Our previous studies had showed that roemerine had very strong anti-cancer activity against the cancer cell lines SGC-7901, HT-29 and MGC-803 with IC_50_ of 0.844 μg/mL, 1.279 μg/mL and 0.631 μg/mL, respectively[23]. The results of the current study showed that roemerine had bactericidal effects against all tested *S*. *aureus* strains with MICs ranging from 32 μg/mL to 64 μg/mL (Table 1). It is possible that roemerine has different modes of action in bacteria and cancer cells. However, roemerine has been found to exhibit cytotoxicity against human umbilical vein endothelial cell with IC_50_ of 43.047 μg/mL[23], which is similar to the MIC against *S*. *aureu*s. Therefore, the structure of roemerine must be further modified to reduce toxicity to human cells.

In summary, our data suggest that roemerine improves the survival rates of septicemic BALB/c mice by increasing cell membrane permeability of *S*. *aureus*. Bacterial resistance is bound to develop against every novel antibiotic due to overuse and abuse of antibiotics and roemerine is no exception. Nevertheless, we believe that the chances of developing resistance to compounds with new structures and novel mechanisms are smaller than that to conventional antibiotics. Taken together, our findings suggest that roemerine may be a good candidate lead compound for treatment of methicillin-resistant *Staphylococcus aureus* infection.
